# A Comparison of Assays for Accurate Copy Number Measurement of the Low-Affinity Fc Gamma Receptor Genes *FCGR3A* and *FCGR3B*

**DOI:** 10.1371/journal.pone.0116791

**Published:** 2015-01-16

**Authors:** Umi Shakina Haridan, Umairah Mokhtar, Lee R. Machado, Abu Thalhah Abdul Aziz, Rafidah Hanim Shueb, Masliza Zaid, Benedict Sim, Mahiran Mustafa, Nik Khairudin Nik Yusof, Christopher K. C. Lee, Suhaili Abu Bakar, Sazaly AbuBakar, Edward J. Hollox, Hoh Boon Peng

**Affiliations:** 1 Institute of Medical Molecular Biotechnology (IMMB), Faculty of Medicine, Universiti Teknologi MARA, Sungai Buloh Campus, Selangor, Malaysia; 2 Department of Genetics, University of Leicester, Leicester, United Kingdom; 3 Department of Microbiology and Parasitology, School of Medical Science, Health Campus, Universiti Sains Malaysia, Kubang Kerian, Kelantan, Malaysia; 4 Department of Medicine, Hospital Sungai Buloh, Jalan Hospital, Sungai Buloh, Selangor, Malaysia; 5 Hospital Raja Perempuan Zainab II, Kota Bharu, Kelantan, Malaysia; 6 Department of Biomedical Science, Faculty of Medicine and Health Science, Universiti Putra Malaysia, Serdang, Selangor, Malaysia; 7 Department of Medical Microbiology, Faculty of Medicine, University of Malaya, Kuala Lumpur, Malaysia; 8 Tropical Infectious Disease Research and Education Centre, Faculty of Medicine, University of Malaya, Kuala Lumpur, Malaysia; University of Bristol, UNITED KINGDOM

## Abstract

The *FCGR3* locus encoding the low affinity activating receptor FcγRIII, plays a vital role in immunity triggered by cellular effector and regulatory functions. Copy number of the genes *FCGR3A* and *FCGR3B* has previously been reported to affect susceptibility to several autoimmune diseases and chronic inflammatory conditions. However, such genetic association studies often yield inconsistent results; hence require assays that are robust with low error rate. We investigated the accuracy and efficiency in estimating *FCGR3* CNV by comparing Sequenom MassARRAY and paralogue ratio test-restriction enzyme digest variant ratio (PRT-REDVR). In addition, since many genetic association studies of *FCGR3B* CNV were carried out using real-time quantitative PCR, we have also included the evaluation of that method’s performance in estimating the multi-allelic CNV of *FCGR3B*. The qPCR assay exhibited a considerably broader distribution of signal intensity, potentially introducing error in estimation of copy number and higher false positive rates. Both Sequenom and PRT-REDVR showed lesser systematic bias, but Sequenom skewed towards copy number normal (CN = 2). The discrepancy between Sequenom and PRT-REDVR might be attributed either to batch effects noise in individual measurements. Our study suggests that PRT-REDVR is more robust and accurate in genotyping the CNV of *FCGR3*, but highlights the needs of multiple independent assays for extensive validation when performing a genetic association study with multi-allelic CNVs.

## Introduction

Copy number variation (CNV) is defined as genetic variation involving a loss or gain of large segments of DNA (typically over 1 kb), a definition that includes simple deletions and duplications [1]. CNV can affect phenotype by altering gene dosage, disrupting coding sequences, or perturbing long-range gene regulation [2], and has been associated with susceptibility to various autoimmune and infectious diseases [3–7].

Fcγ receptors function as cell surface receptors for the Fc region of IgG, are found on the surface of natural killer cells, monocytes, neutrophils, and mast cells, and play a critical role in immunity. In humans, there are three main types of Fcγ receptors, high-affinity FcγRI and low affinity FcγRII and FcγRIII [8]. They bind to IgG-antigen immune complex and initiate either inhibitory or activating responses within the cell [9]. FcγRII receptors are encoded by *FCGR2A*, *2B*, *2C* and FcγRIII receptors are encoded by *FCGR3A* and *FCGR3B* respectively, and these genes are located as a cluster at 1q23.3. *FCGR3A* and *FCGR3B* encode FcγRIIIA and FcγRIIIB respectively, which are different from each other both in their attachment to the cell surface and in their expression pattern: Fc**γ**RIIIA has a transmembrane region and is expressed on natural killer cells, and Fc**γ**RIIIB is attached to the cell membrane by a glycophosphoinositol anchor and is expressed primarily on neutrophils [10]. *FCGR3A* and *FCGR3B* are paralogous genes each on an 82 kb segmental duplication which is ~98% identical at the DNA level. *FCGR3B* carries the two common alleles of the human neutrophil antigen HNA1 that differ by four amino acid substitutions: HNA1a and HNA1b [11]. Recurrent non-allelic homologous recombination between the two segmental duplications generates CNV within populations affecting both *FCGR3A* and *FCGR3B* [12–14].

The CNV of *FCGR3B* has been reported to be associated with susceptibility to a number of autoimmune diseases including systemic lupus erythematosus (SLE), organ-specific autoimmunity, and rheumatoid arthritis [14–16]. However, genetic association studies for multi-allelic CNV remains problematic as spurious copy number calls could lead to false association thus yield inconsistent replications [17–19]. This is predominantly due to lack of robust and accurate methods in assaying such genetic variation. Therefore in this paper, we compare three typing assays: paralogue ratio test-restriction enzyme digest variant ratio (PRT-REDVR) [13], real-time qPCR [16] and Sequenom MassARRAY (Sequenom Inc. Brisbane, Australia).

## Materials and Methods

### Ethical approval and subject recruitment

This study obtained approval from the Research and Ethics Committee of Universiti Teknologi MARA (UiTM) [600-RMI (5/1/6)], Universiti Sains Malaysia (USM) [USMKK/PPP/JePeM [211.3.(6)] and the Ministry of Health [NMRR-09–1128–4211]. Informed and written consent were obtained from the individuals for the study of host genetic susceptibility to dengue fever. A total of 237 dengue patients were recruited from Hospital Kota Bharu (HKB) and Hospital USM. A total of 3 ml peripheral blood was collected for each recruited individual.

### DNA extraction

The QiaAMP Blood Mini Kit (Qiagen, Germany) was used to extract DNA from 200 μl of peripheral blood sample according to the manufacturer’s protocol. Concentration and purity of the extracted DNA were determined by spectrophotometer Nanodrop ND-1000. The quality of DNA was confirmed by electrophoresis on a 1% agarose gel, followed by SyBr safe staining to ensure that the DNA samples were not degraded based on the clear single band generated.

### Real-time quantitative PCR (qPCR)

The primer sequences for the target gene *FCGR3B* and the reference gene *FOXP2* were obtained from Fanciulli et al. [16]. A total of 5 μl (10 ng/µl) genomic DNA was amplified in a reaction mixture containing 12.5 μl iQ SyBr Green Supermix (BioRad), 1 μl (7 μM/μl) of each of forward and reverse primers, and made up to total volume of 25 μl with ddH_2_O. Cycling conditions were 95˚C for 3 min, and then 40 cycles of 95˚C for 30 s, followed by 60˚C for 15 s and 72˚C for 30 s. The *FCGR3B* primer sequences were: Forward: 5′-CACCTTGAATCTCATCCCCAGGGTCTTG-3′ and Reverse: 5′-CCATCTCTGTCACCTGCCAG-3′. The amplification was carried out using BioRad CFX96 Touch Real-Time PCR Detection System.

The efficiency of the assay was determined by the generation of a standard curve: a series of five-fold dilutions of a single genomic DNA sample from 50 ng/μl to 0.08 ng/μl (10 ng to 0.016 ng in each qPCR). All reactions were run in triplicate. For a high quality assay, amplification should be linear across the entire dilution series with amplification efficiencies between 90–110%. Normalization to the control gene Forkhead Box P2 (*FOXP2*) (Forward: 5′-TGACATGCCAGCTTATCTGTTT-3′ and Reverse: 5′-GAGAAAAGCAATTTTCACAGTCC-3′) [16] was used to give an estimate of copy number. Copy number of the target sequence in each test sample was determined by using the comparative CT (2-CT) approach. The relative copy number of the target gene in each test sample in relation to the reference gene was determined by relative quantification through the comparative CT (ΔΔCT) calculation method as previously mentioned by Livak and Schmittgen [20]. Assumption of exact doubling of the target sequence was made in this method. After normalisation to the median value of all samples in the assay, which was assumed to represent a diploid copy number of 2, deletions of *FCGR3B* were called for samples with a value <1.5, and duplications of *FCGR3B* were called for samples with a value >2.6. These values were chosen based on clustering of raw copy number values of the entire sample set.

### Sequenom MassARRAY genotyping

Sequenom MassARRAY uses the primer extension approach for both relative, and absolute determination of CNV calls [21]. For both *FCGR3A* and *FCGR3B* three assays were designed at the 3-prime, 5-prime and centre of each gene, targeting a single nucleotide variant (SNV) that distinguished the two paralogues [22] (Table 1). In effect, these SNV sites were being used for paralogue-specific quantification, hence the relative signal from each paralogue was investigated. All test samples were assayed with one multiple primer master mix, and primer extension PCR was performed. The extended products were analysed by SEQUENOM MALDI-TOF mass spectrometry.

**Table 1:** Primer sequences and single nucleotide variation (SNV) identified for *FCGR3A/B* in Sequenom MassARRAY.

**Primer**	**Sequence**	**SNV identified**	
		**3A**	**3B**
FCRG3A/B—centre	5′-ACGTTGGATGTGGTTGTAGGTGGACATCTC	**T**	**C**
	3′-ACGTTGGATGCTGACATGGTCTTCACTCTC		
FCGR3A/B—3′	5′-ACGTTGGATGAGATATCCGGAGCCCTAAAG	**A**	**A**
	3′-ACGTTGGATGTGACAGAGATGGGTGGAGG		
FCGR3A/B—5′	5′-ACGTTGGATGCAGTAGTACATTTAGTATTGG	**C**	**T**
	3’-ACGTTGGATGAAAACCACCTTTTCTGCTTC		

There are two steps in Sequenom MassARRAY allowing the inference of genomic copy number. The first stage relies on comparison of an amplicon for the region of interest with a known amount of a competitor DNA amplicon of known genotype, using primer extension and mass spectrometry to quantify the amount of each variant at a particular SNV. Comparison of the ratio of the genomic amplicon with the competitor DNA provides information about the copy number of the region of interest. The second stage relies on a comparison of the amount of amplicon for the region of interest and the amount of a control amplicon where diploid copy number is assumed to be two, co-amplified from the same genomic DNA. Three control regions were included in the study: i) chr11: 31,700,000–31,880,000; ii) chr6: 43,864,065–43,904,064; iii) chr7: 113,800,000–114,150,000, reference coordinates based on genome assembly build hg18. Approximately 100–120 bp of oligonucleotide sequence corresponding to the control and test regions was synthesized and titrated by serial dilution to optimise the iPLEX experiment (Sequenom) for equal peak sizes for the control regions and test region. The 2N controls were used to normalize intra-assay sample to sample loading variation, and the CNV of interest against a normal control.

The details of analysis of copy number calls for MassARRAY are described in the Sequenom Quickguide [21]. In brief, absolute copy number was calculated from the logEC50 value generated via the titration series in Sequenom QGE software provided by Sequenom Inc. The EC50 value is the point at which the peak areas of the test DNA and the competitor DNA are equal, representing a 1:1 concentration of the molecules in the reaction (ratio 1:1). This point is defined as the effective concentration required for obtaining 50% of the maximal effect (EC50). Subsequently the absolute copy numbers obtained were further normalized as was done in the qPCR mentioned earlier.

### Paralogue Ratio Test (PRT)

The PRT-REDVR assays were carried out as described according to Hollox et al. [13]. Briefly the amplification of 10 ng of DNA was performed in a final volume of 10 μl, with 0.5 μM forward primer and 0.5 μM FAM- or HEX- labelled reverse primer, in a reaction buffer. The primer sequences used are shown in Table 2. The two amplifications were carried out (with FAM- or HEX- labelled primer respectively), to allow detection and co-electrophoresis of the amplicons on the capillary electrophoresis. This allows internal calibration of each experiment. Products were amplified using 30 cycles of: 95˚C for 30 s, 56˚C for 30 s and 70˚C for 30 s followed by single chase of 56˚C for 1 min then 70˚C for 20 min to reduce levels of single-stranded DNA products. After the PCR cycle, 1 µl of a 10% to 20% dilution of each PCR product was added to 10 µl deionized formamide, and analysed by electrophoresis on an ABI 3100 Genetic Analyzer (Applied Biosystems, Warrington, UK), with an injection time of 30 s.

**Table 2:** Primer sequences for *FCGR3* PRT assay.

**Primer orientation**	**Sequence**
FCGR3B_Forward (HEX)	5′-ATGATCTGGCCCTGAAACTC-3′
FCGR3B_Forward (FAM)	5′- ATGATCTGGCCCTGAAACTC -3′
FCGR3B_Reverse	5′-TGAGTTCAAGAAAGCAGTTG-3′

The respective PCR amplicons sized 67 bp corresponding to chromosome 1, and 72 bp corresponding to chromosome 18, were recorded for both FAM- and HEX- labelled products. The ratio of the areas under the 67 bp peak and the 72 bp peak was compared, and the results were accepted if the coefficient of variation (standard deviation divided by the mean) was <0.15. The mean of the FAM and HEX ratio was used in further analysis.

Both the mean ratios and 9 reference standards with known copy numbers obtained from Human Random Control DNA (European Collection of Cell Cultures) were used as the calibration for each experiment, and the resulting linear regression was used to estimate the copy number of FCGR3 for the samples studied, as described in [13].

### Restriction Enzyme Digest Variant Ratio (REDVR)

Two REDVR assays were used in this study: one distinguishes variant from *FCGR3A* and *FCGR3B* (c.733C>T, corresponding to the arginine to stop codon change that defines *FCGR3B*) and the other distinguishes neutrophil antigens HNA1a and HNA1b (g.147C>T) [13]. Amplification of two regions in duplex was carried out using primer sequences as shown in Table 3 with concentration of 0.5 μM and the conditions described above, except with an annealing temperature of 53˚C: 2 μl of PCR product was digested with 10 units of *Taq^α^*I restriction enzyme (New England Biolabs) in 50 mM Tris-Cl (pH 7.9 at 25˚C), 100 mM NaCl, 10 mM MgCl_2_,1 mM dithiothreitol in a final volume of 10 μl for 4 hours at 65˚C. Digested products were analysed with capillary electrophoresis on an ABI 3100 Genetic Analyzer (Applied Biosystems) and analysed with GeneScan software (Applied Biosystems). Mean ratios of the product were used along with the reference standard for experimental calibration and the result was used to estimates copy number calls.

**Table 3:** Primer sequences for REDVR assay.

**Primer_orientation**	**Sequence**
38_Forward (FAM)	5′-AAGACTGAGCCACCAAGCAT-3′
38_Reverse	5′-CTCCCTGGCACTTCAGAGTC-3′
234_Forward	5′-TTTTGCAGTGGACACAGGAC-3′
234_Reverse (HEX)	5′-GGGTTGCAAATCCAGAGAAA-3′

The primer pair 38 distinguishes *FCGR3A* from *FCGR3B* while the primer pair 234 distinguishes the HNA1a region from HNA1b.

The PRT analysis for copy number call was performed in combination with the REDVR analysis, using a maximum likelihood approach described previously [13]. PRT produces the sum of the copy number calls for *FCGR3A* and *FCGR3B* genes whilst the REDVR estimates the copy numbers of *FCGR3A* and *FCGR3B* based on the ratio determined [13]. Analyses were performed using Microsoft Excel unless otherwise stated.

## Results and Discussion

**Figure 1:**
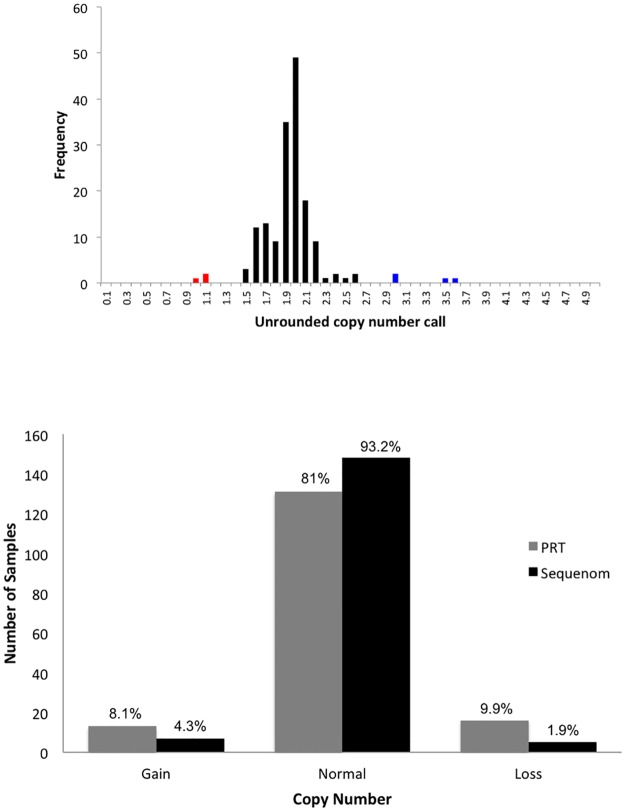
Distribution of raw unrounded copy number estimates of *FCGR3A*. A, Analysis was carried out using 160 samples genotyped with Sequenom. Unrounded copy number estimates are in bins of 0.1, with the count of each bin displayed on the y-axis. Blue indicates samples that were called as duplications, and red samples that were called as deletions. The red bars indicate copy number loss (CN<2); black bars indicate copy number normal (CN = 2); blue bars indicate copy number gain (CN>3). **B**, Distribution frequency of absolute copy number call for the samples genotyped with Sequenom MassARRAY and PRT-REDVR shows a higher rate of copy number normal (CN = 2) being called by Sequenom.

**Figure 2:**
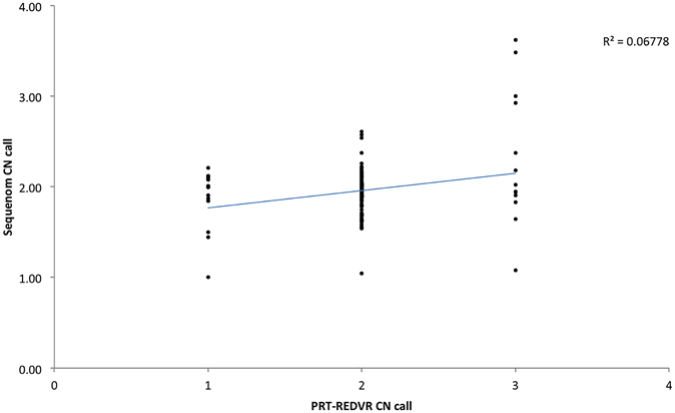
Scatterplot showing 160 matched samples for *FCGR3A* CNV estimated by PRT-REDVR and Sequenom. Sequenom values represent raw copy number, PRT-REDVR values represent integer copy numbers. Each point indicates sample being called by both Sequenom and PRT-REDVR. The blue line indicates the trendline, suggesting an general concordance of the two platforms.

We first assessed the distribution of the raw copy number calls for *FCGR3A* from Sequenom (Fig. 1A). Distinct clustering was observed around the integers, indicating relatively high consistency of this assay in *FCGR3A*. 81% (101/161) of the absolute copy number from Sequenom were in agreement with PRT-REDVR, but the Sequenom data tended to be skewed towards a diploid copy number of two (Fig. 1B; S1 Table). A higher rate of inconsistency between these two assays was observed with higher copy number (Fig. 2).

**Figure 3:**
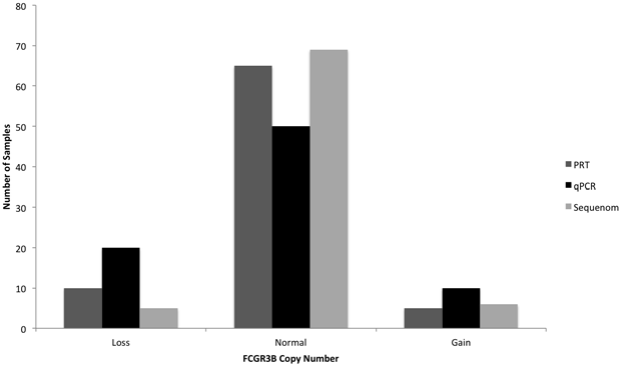
Distribution frequency of absolute copy number call of *FCGR3B* for the samples genotyped with all three different assays respectively. Analysis was carried out with 80 samples genotyped with PRT-REDVR, Sequenom and qPCR. Loss, copy number < 2; normal, copy number = 2; gain, copy number > 2.

**Figure 4:**
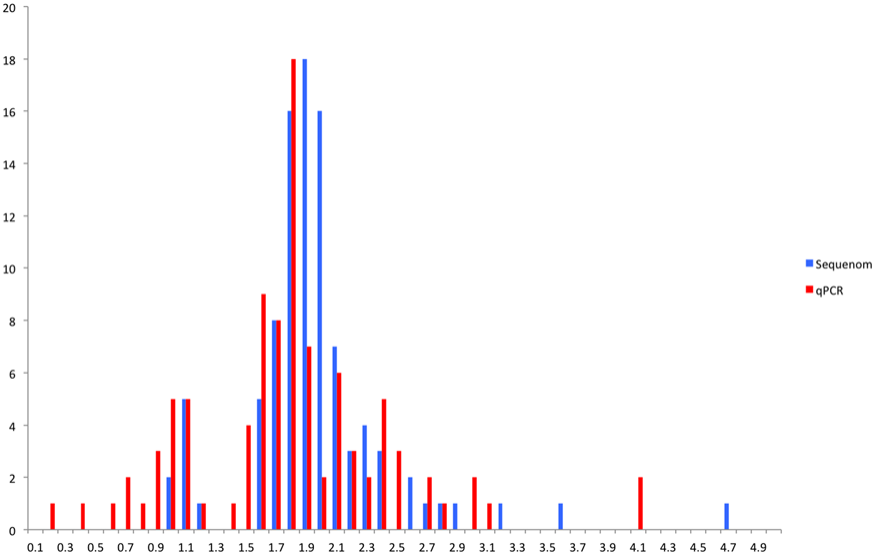
Distribution of unrounded copy numbers called by qPCR and Sequenom for *FCGR3B*. Analysis was carried out using 100 samples genotyped with both qPCR and Sequenom. Unrounded copy number estimates are in bins of 0.1, with the count of each bin displayed on the y-axis.

**Figure 5:**
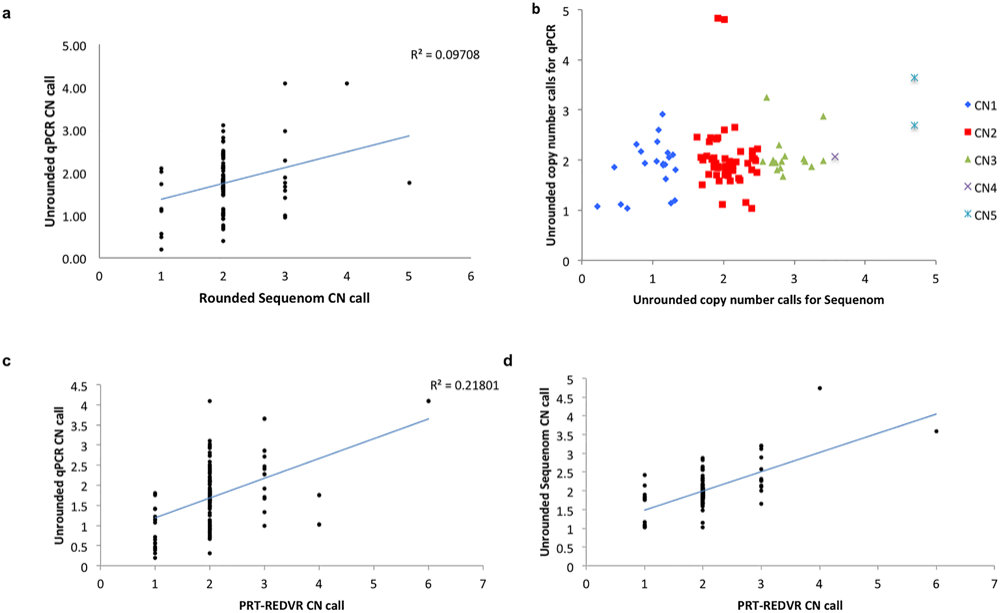
Scatterplot showing all 99 samples matched for *FCGR3B* CNV with PRT-REDVR, Sequenom and qPCR. **A.** qPCR vs Sequenom. qPCR values represent raw copy number, Sequenom values represent absolute copy numbers, **B.** Sequenom vs qPCR. Both Sequenom and qPCR raw copy number values are presented, showing a low concordance rate between the two platforms, **C.** qPCR vs PRT-REDVR. qPCR values represent raw unrounded copy number, PRT-REDVR values represent absolute copy number; and **D.** Sequenom vs PRT-REDVR. Sequenom values represent raw unrounded copy numbers, PRT-REDVR values represent absolute copy number. The broader scater plots of qPCR signal intensity show wider variability of copy number calls, suggesting more copy number calls from qPCR were discordant with the Sequenom and PRT-REDVR. D exhibits a more clustered plot indicating higher agreement of CN calls between Sequenom and PRT.

Next, we analysed the copy number calls for *FCGR3B* with the sample genotyped with all three assays namely, qPCR, Sequenom and PRT-REDVR (N = 80). In general, copy number called by qPCR showed a greater variability (gains or losses) (Fig. 3), and relatively broader distribution compared to Sequenom (Fig. 4), suggesting lower specificity of qPCR, in line with the previous report [23]. The performance of the assays was assessed by matching the data for PRT, qPCR and Sequenom (Fig. 5; S2 Table). qPCR revealed a lower consistency with the two assays (Fig. 5A—C); whereas the raw copy number call from Sequenom was in higher agreement with PRT-REDVR (Fig. 5D). We observed that 78.2% of the copy number calls for *FCGR3B* were in agreement between PRT-REDVR and Sequenom; while qPCR showed a lower concordance rate with PRT-REDVR (60.1%) and Sequenom (75.4%) (Table 4; S2 Table). The discordance increased with the increase of copy number calls. The concordance rate for all the three assays though, has been relatively poor (57.3%). This observation agrees with the report from the previous study [23]. CNV typing was repeated on the 10 of the discrepant samples by qPCR and PRT- REDVR and showed that six of these samples the discrepant measurements are reproducible. We speculate that this could be due to small repeat specific deletions, or perhaps reflect single nucleotide variation underneath oligonucleotide primers that reduce or abolish amplification from one repeat [13].

**Table 4:** Percentage of concordant results of *FCGR3B* CN calls between the three different assays.

	**PRT-REDVR**	**qPCR**
**PRT-REDVR**	-	-
**qPCR**	60.1%	-
**Sequenom**	78.2%	75.4%

Collectively, our analyses strongly suggest that, i) the higher copy number, the greater discrepancy in CNV estimation between assays; and ii) neither Sequenom nor qPCR assay is suitable for multi-allelic copy number variation genotyping.

**Figure 6:**
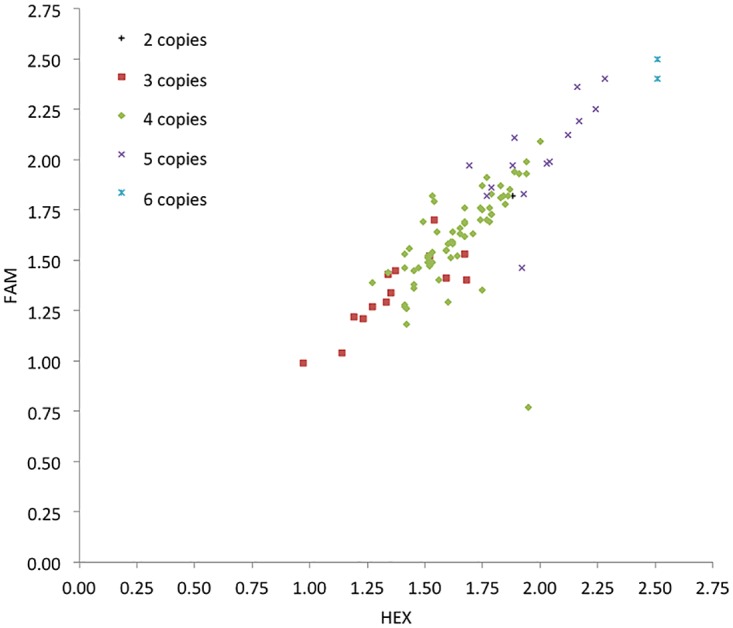
Analysis of raw copy number quantification data for PRT. Correlation between individual duplicated PRT copy number. Individual results from FAM-labelled (Y-axis) versus Hex-labelled (X-axis) representing internal replication of the assay, are plotted for 99 samples. Colour coded according to the integer copy number of each samples as estimated in REDVR.

**Figure 7:**
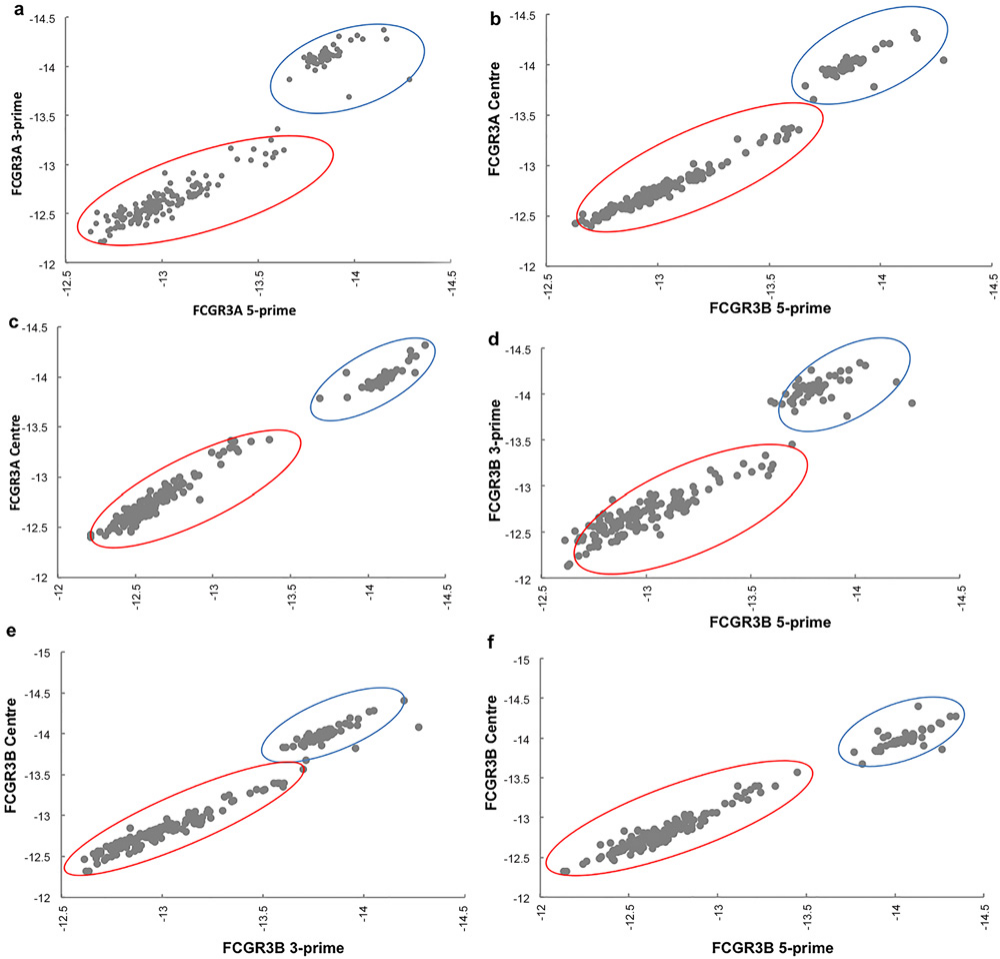
Analysis of raw quantification data (logEC50) for Sequenom MassARRAY. Correlation between raw individual copy number calls from the probes designed at 5′-, 3’-, and centre regions of the genes *FCGR3A* (A, B, and C) and *FCGR3B* (D, E, and F). Note that there is a clear seperation of 2 cluster plots indicating batch effects, as circled, due to different experimental batches. **A**, **3’ vs 5′; B, 3’ vs centre;** and **C, 5′ vs centre**; **D**, **5′ vs 3’; E, 3’ vs centre;** and **F, 5′ vs centre.** A and D, correlation between the 5- and 3- prime probes were slightly affected, probably due to the probes distance.

To assess the quality of the copy number calls from PRT, we showed that both FAM- and HEX-labelled PRT duplicates were highly correlated, except for one outlier (Fig. 6). Although the clusters overlap, final copy number calls are made using information both from the PRT data and the two REDVR assays. The raw Sequenom data (logEC50) generated from the three distinct probes did correlate well with each other, but was slightly affected by the distance between probes especially with the highly copy number variable *FCGR3B* (Fig. 7). However the advantages of having three probes outweighs the disadvantages. Another potential drawback of the Sequenom assay is the batch effect observed (Fig. 7), which indicates that large changes in absolute copy number can be generated by batch effect alone, although comparison with the control probes may reduce this effect in the relative copy number estimates.

PRT is a PCR-based assay using a single pair of primers to simultaneously amplify two specific products in a single reaction: one from a single-copy reference locus and the other from the copy variable test locus of interest–in this case, the *FCGR3* region. The copy number of the test locus is then estimated from the ratio of test to reference PCR products.

PRT relies on co-amplification of the test and reference locus using the same primer pair, with test and reference distinguished by a small deletion, subsequently differentiated and quantified using capillary electrophoresis. The primer pairs were designed in such a way that that it amplifies a unique reference locus on chromosome 18 [13]. The test and reference PCR products can be distinguished by a small difference in size [13]. One limitation of this PRT assay is that its development requires the presence of paralogue DNA sequence in the CNV regions to allow comparison of ratio between reference and test locus, which at times not applicable to many CNV loci. In addition, it only amplifies copies of a generic *FCGR3*, owing to the fact that this region shows up to 98.3% sequence homology, posing a challenge in designing primers specific for *FCGR3A* and *FCGR3B* CNV genotyping. Therefore, a multiplex REDVR assay was used to amplify across the nucleotide which changed an arginine in *FCGR3A* to a stop codon in *FCGR3B*, and a nucleotide that distinguished HNA1a from HNA1b. The a/b variant can be distinguished by digestion using *Taq^α^I*. The ratio of 3A:3B and HNA1a:HNA1b also provides extra information on copy number helping distinguish copy numbers.

In both PRT and REDVR, inter-experiment calibration was corrected by running known controls showing high agreement for total copy number between methods. This highlights the advantage of this assay whereby it generates more reliable integer copy number calls with inter-validation whilst easing the interpretation.

Our study suggests that qPCR could potentially introduce false-positive calls, therefore CNV association studies based on qPCR should be counter validated. Indeed, many studies showed contradictory findings on copy number and association with disease development, for instance *CCL3L1* in HIV [24], and *DEFB4* in Crohn’s disease [19, 25]. The disadvantage of qPCR though is common across copy number assays and this is expected in view of the principle of CNV and the chemistry of qPCR amplification. The absolute values also vary usually upon repetition since qPCR is technically demanding. This again is a common phenomenon observed in most qPCR assays [26].

Reports of CNV genotyping based on Sequenom have been relatively lacking. However, more conservative calls in this assay potentially dilute true positive calls of CNV.

In summary, we suggest that qPCR assay is not suitable for the use in large scale case-control association studies for multi-allelic genes like *FCGR3*. On the other hand, PRT-REDVR presents a relatively more reliable result as compared to both qPCR and Sequenom MassARRAY [24]. The sequence requirements for PRT assay development mean that it is limited to certain sequences, and an assay cannot necessarily be designed for a small sequence region, for example, one specific exon. However, the precision and accuracy of PRT-REDVR are equivalent to those of MLPA and MAPH, and it has the advantage of potential high-throughput analysis with the small amount of DNA required as of PCR-based methods [27]. Advantages further include the robustness of assay, small amount of DNA, though potentially introducing false negative result, running cost, and feasibility of equipment. The DNA integrity though is a major determining factor for the specificity and robustness of an assay. Independent assay such as microsatellite therefore, should warrant the informativeness of this technology [13].

## Supporting Information

S1 Table Unrounded and absolute copy number calls for FCGR3A from Sequenom and PRT-REDVR. (XLSX)

S2 Table Unrounded and absolute copy number calls for FCGR3B from qPCR, Sequenom and PRT-REDVR. (XLSX)
